# Solving the Evidence Interpretability Crisis in Health Technology Assessment: A Role for Mechanistic Models?

**DOI:** 10.3389/fmedt.2022.810315

**Published:** 2022-02-24

**Authors:** Eulalie Courcelles, Jean-Pierre Boissel, Jacques Massol, Ingrid Klingmann, Riad Kahoul, Marc Hommel, Emmanuel Pham, Alexander Kulesza

**Affiliations:** ^1^Novadiscovery SA, Lyon, France; ^2^Phisquare Institute, Transplantation Foundation, Paris, France; ^3^European Forum for Good Clinical Practice, Brussels, Belgium

**Keywords:** modeling and simulation (M&S), mechanistic evidence, drug development, health technology assessment (HTA), stakeholder engagement (SE), mechanistic models

## Abstract

Health technology assessment (HTA) aims to be a systematic, transparent, unbiased synthesis of clinical efficacy, safety, and value of medical products (MPs) to help policymakers, payers, clinicians, and industry to make informed decisions. The evidence available for HTA has gaps—impeding timely prediction of the individual long-term effect in real clinical practice. Also, appraisal of an MP needs cross-stakeholder communication and engagement. Both aspects may benefit from extended use of modeling and simulation. Modeling is used in HTA for data-synthesis and health-economic projections. In parallel, regulatory consideration of model informed drug development (MIDD) has brought attention to mechanistic modeling techniques that could in fact be relevant for HTA. The ability to extrapolate and generate personalized predictions renders the mechanistic MIDD approaches suitable to support translation between clinical trial data into real-world evidence. In this perspective, we therefore discuss concrete examples of how mechanistic models could address HTA-related questions. We shed light on different stakeholder's contributions and needs in the appraisal phase and suggest how mechanistic modeling strategies and reporting can contribute to this effort. There are still barriers dissecting the HTA space and the clinical development space with regard to modeling: lack of an adapted model validation framework for decision-making process, inconsistent and unclear support by stakeholders, limited generalizable use cases, and absence of appropriate incentives. To address this challenge, we suggest to intensify the collaboration between competent authorities, drug developers and modelers with the aim to implement mechanistic models central in the evidence generation, synthesis, and appraisal of HTA so that the totality of mechanistic and clinical evidence can be leveraged by all relevant stakeholders.

## Introduction

Health technology assessment (HTA) is a systematic and multidisciplinary process that summarizes medical evidence, social and economic impact, and ethical issues related to the use of health technology. HTA addresses both the direct and intended effects of this technology, as well as its indirect and unintended consequences—with the goal of informing decision making. A major feature of the collective output of a HTA process is the reimbursement by the health insurance system of the medical product (MP).

In general, two levels of decision-making regarding health care should be informed by HTA: (1) for the community—is the MP worth giving to the population, and could it be more or less beneficial for a group in the population? (2) for an individual: will a particular patient benefit from the MP, and if yes to what extent?

HTA seeks to couple the available evidence on the MP and the disease with the decision-making process itself, and thus has similarities to evidence-based health care and evidence-based policymaking (1). By evidence, one should understand a comprehensive record of knowledge and data collected in clinical trials (of which randomized, placebo-controlled studies are the gold standard), observational studies and from various sources relating to patient health status and/or the routine delivery of health care (often referred to as “real-world data,” RWD). One could say that HTA interprets clinical data from a real-world perspective by considering the realistic epidemiology of the disease and the full range of standard of care options (available to the population of interest). For a given MP (we focus on new drugs in this Perspective), the first round of assessment occurs during the review of the market authorization (MA) application by the regulators, e.g., FDA (Food and Drug Administration) or EMA (European Medicines Agency) for safety and efficacy. Given that the evidence included in these applications is generated throughout several years of development, key stakeholders could and should synergize and could streamline evidence generation and assessment from the beginning (2). Non-RCT data such as observational study data or RWD might bear relevant and additional information about safety, effectiveness, and cost effectiveness of MPs at potentially a larger scale. However, issues with identification, access, quality, representativeness, and heterogeneity of such data are limiting their practical applicability in HTA (3, 4).

The COVID-19 pandemic has disrupted global healthcare systems and created significant challenges for the HTA and payer communities (5). The COVID-19 pandemic has clearly shown where evidence generation, synthesis, assessment, and decision making are limited: (a) the typical bench-to-bedside timeframes of several years are simply unacceptable in a pandemic context; (b) clinical trial evidence collected in “emergency mode” suffers from increased uncertainty regarding the expected treatment effect, outcomes and costs (6); (c) the diversity of national policies and their frequent changes make it hard to come to conclusions on ethical and societal issues and raise barriers for patients to fully capture and understand the impact of a new MP on their life.

Especially in light of the COVID-19 pandemic, the importance of the assessment for the individual cannot be underestimated. While some patients do not suffer from any symptoms, others do not survive, or are affected on long timescales. Clinical data on COVID-19 prophylaxis and treatment currently under-represents the individual course of the disease due to the diversity and time dependency of the interactions between the virus and the patients' bodies. Here, the inherent limitation of HTA—being centered around population-based approaches—is aggravated. Issues related to better guiding economic evaluation of personalized medicine interventions—e.g., how study questions are developed, how populations are characterized, how comparators are defined, how effectiveness is evaluated, how outcomes are valued and how resources are measured (7)—need urgently to be addressed for the assessment of MPs related to COVID-19.

The COVID-19 pandemic has also raised the bar for communication around HTA. There has been divergence of opinion among international HTA agencies on how to deal with evidence for early COVID-19 treatments (8). This divergence and lack of transparency about the reasoning behind the assessments during this unsettling period have triggered public unease and skepticism with HTA as a whole.

As a response to the urgency to address these challenges, we wish to advocate using mechanistic models to bridge clinical MP development and HTA thanks to their capability for evidence generation, synthesis, and stakeholder communication alike.

## Challenges for Health Technology Assessment

A key issue for HTA of a new MP is the number of limitations regarding the representativeness and validity of the evidence that is available. For conclusions useful for patients and public health, more quantitative knowledge and valid answers to questions need to be found (Table 1).

**Table 1:** Examples of how published (mechanistic) models rooted in the clinical development space (model informed drug development, MIDD) could address uncertainties in new medicinal product assessment reports.

**Uncertainty not completely addressed in competent authority assessment report**	**Example use of MIDD relevant to address uncertainty potentially also during HTA**
What is the optimal dosage in the clinical context?	Physiologically based pharmacokinetic models can investigate dosing-regimens relevant for regulatory review and product labels (9) and can also mimic real-life adherence to prescribed treatment regimens (see also below) or pharmacology-relevant characteristics of special populations as well as drug-drug interactions.
What is the duration of the effectiveness, especially with chronic use of a treatment?	Mechanistic models can predict the long-term disease progression by extrapolation of shorter-term findings under the constraints of how the components of the system function (and these constraints convey biological plausibility by design). An example is the use of a mechanism-based disease progression model for comparison of long-term effects of pioglitazone, metformin, and gliclazide on disease processes underlying Type 2 Diabetes Mellitus (10). Another example is prediction of long-term outcomes by short-term marker data as demonstrated by a semi-mechanistic approach in context of osteoporosis treatment (11).
What is the efficacy for relevant clinical outcomes?	Mechanistic models combined with pharmacometric approaches can translate findings for one outcome to a range of other outcomes. An example of survival modeling on the back of a mechanistic description is the modeling framework for CD19-Specific CAR-T cell immunotherapy using a quantitative systems pharmacology model (12).
What is the size of the clinical effect dependent on patient characteristics and extrinsic factors?	Data-driven modeling techniques can capture correlation within clinical data. Describing the clinical effect of a drug can also be based on mechanistic considerations. Such models either (a) link disease phenotypes to increasingly granular mathematical representations of pathophysiologic processes (top-down approach) or (b) derive functional, computable cellular networks from the molecular building blocks of genes and proteins to elucidate the impact of pathologic or therapeutic alterations on network operating states and hence clinical phenotype (bottom-up) [see (13)]. In this way, functional relationships can explain the found correlations and can be used for quantitative analysis of the effect size and the causality dependent on intrinsic and extrinsic factors.
What is the difference in effect when compared head-to-head to other comparators?	Mechanistic modeling is a commonly used tool to explore treatment combinations in immuno-oncology [see for example (14)] which can enable head-to-head comparisons. A mechanistic approach with clinical trial simulation can provide model-based meta-analysis which can ameliorate indirect comparison of clinical data (15).
What is the efficacy compared to placebo or the standard of care, when controlled studies are hard to conduct?	For comparative effectiveness research, data from a control arm is needed. When such control arm is unfeasible (for example because of ethical reasons), external or synthetic control data may be an avenue to put uncontrolled clinical data into a controlled setting, but mitigation of the risk of bias needs adjustment techniques. Mechanistic modeling can quantitatively predict the effect of an intervention on a clinical outcome as a function of patient characteristics and extrinsic factors, on a single patient level. These features render mechanistic models promising to set up unbiased synthetic control arms [SCA, see (16)].
What is the effect of real-life compliance on efficacy?	Explicit simulation of administration adherence can be coupled with pharmacokinetic models. One example is the simulation of adherence patterns using Markov Chains for trial design (17, 18).
What is the distribution of responders in the target population?	Predicting individual response to treatments needs the convergence of large-scale mechanistic models [e.g., in cancer pathways (19)], appropriate responder profiling framework and cost-effectiveness analysis [for example the Effect Model approach, see (20, 21)]
What is the size of the benefit at the population level?	Mechanistic models providing clinical outcome estimates can be used on the entire population level to predict effectiveness, given that adapted metrics are used (22)
What is the long-term safety and what impact does the occurrence of rare side effects have over long-term use?	The combination of quantitative systems toxicity (23) with organ (e.g., cardiac, and renal) impairment (24) in frame of disease progression modeling (25) can be used to simulate long term safety aspects of a treatment from a mechanistic point of view

*Emphasis is put on mechanistic models*.

### Randomized Clinical Trials (RCTs) Deliver a Binary Answer to a Binary Question

A first reason for the limited use of data generated during development lies in the results provided by randomized clinical trials (RCT) which are the gold standard for clinical evidence. An RCT is an instrument built to determine if the new treatment is effective or not by statistical testing. The frequentist inference paradigm (26) is still today's standard method in RCT despite the advent of innovative trial designs and analysis techniques [i.e., Bayesian (27)] but can limit drastically the interpretation of the efficacy tested in the trial (26, 28). In addition, the fact that statistical models are not designed to look for causality—but only to identify correlations available in the data—prevents a quantitative appraisal of the MP efficacy tailored to patient profile (20).

### RCT Data Reflects Benefit of the Population and Not of the Individual

A second limitation in HTA is the fact that currently population (and sometimes stratified) medicine is pursued during clinical development while for HTA, the benefit for individual patient (groups) becomes important. RCTs, done either separately for different strata for the population or analyzed for different subgroups of one larger study population are currently the only tool available to “individualize” a MP efficacy estimate. As it is the central focus of an RCT to robustly estimate the average effect in a given population, cannot be obtained easily and hence, detailed information at patient level and the mean estimated effect is “applied equally” to each patient. Frequently, patients enrolled during clinical development are not entirely representative of the future target population because of the way they are selected to enter the trials. And they are furthermore limited in number and diversity. Reliably quantifying the effect for individuals from this evidence is therefore limited as well.

The advent of personalized medicine puts the “mean efficacy” approach in question (7) and calls for a paradigm shift of how efficacy should be considered for market authorization (MA)—and market access.

### High Quality Data Exceeding the Scope of Market Authorization Is Scarce

For sponsors, there are increased barriers to conducting randomized trials after registration. Availability of a treatment with proven efficacy may pose ethical problems for placebo-controlled trials. Additional information about the effect of a treatment often needs to rely on observational studies and RWD (for example registers, patient records). The fact that RWD contains routinely collected information and low accessibility but high heterogeneity of data (29, 30) does not easily reveal the detailed and true epidemiological status of a disease or the effect of an intervention in the population. Even with the additional use of RWD, it remains difficult to derive an overview of the long-term and real-life impact in the clinical practice necessary for the HTA exercise.

In summary, gold-standard evidence for HTA (RCTs) can be regarded as more qualitative than quantitative, it has a domain of validity restricted to the context tested in clinical trials during clinical development and does not answer a number of important questions (see Table 1). It is not always possible to collect enough high-quality observational data and RWD to fill the gaps. In view of these challenges, and even more so when there is a strong, urgent, unmet therapeutic need (as today—facing the COVID-19 pandemic), HTA agencies are faced with a difficult dilemma: They can assess and position themselves on the basis of uncertain evidence (risk of misjudgement) or wait for more solid evidence (risk of delaying the access to a potentially effective product for patients with progressive disease or in treatment failure). This situation advocates to make better use of the “totality of evidence” generated during development.

## Advent of the Mechanistic Approach in Model Informed Drug Development

Model-informed drug development (MIDD) applies drug exposure-based, (systems) biological and statistical models derived from preclinical and clinical data sources to inform drug development and decision-making (31). It integrates information from diverse data sources to decrease uncertainty and lower failure rates, and to develop information that cannot or would not be generated experimentally. The most widespread fields of application within MIDD are pharmacokinetics and pharmacodynamics and dose-response relationship modeling for dosing-regimen explorations as well as trial simulation for design optimization.

Within MIDD and regulatory decision making, a new set of models is emerging (32, 33). These models are based on knowledge with theoretical rules describing known mechanisms (called mechanistic models^1^). Within the family of mechanistic models physiologically based pharmacokinetic modeling (PBPK) adopts a mechanistic approach to describe what the body does to the drug and quantitative systems pharmacology (QSP) models aspire to capture what the drug does to the marker, organ, or clinical outcome. As opposed to data driven models, mechanistic ones describe known or hypothesized mechanisms at a smaller scale so that the higher scale behavior emerges (34). In most mechanistic models the equations describe functional relationships between molecules, cells, or organs. The choice of the used equations and their parameters is informed through systematically reviewing and curating the available biomedical knowledge about the process of interest, and in turn, each component of the model (variable state, parameter, and equation) can be unequivocally justified by a corresponding piece of knowledge in the literature (or other considered source of knowledge)^1^. The equations often come in the form of systems of ordinary differential equations (ODEs) that can describe coupled dynamics of the entities in the biological system of interest (but also other approaches such as partial differential equation systems or agent-based models exist). The covered composition of biological entities and scale of the description such as molecules, cells, organs, or the whole organism can vary depending on the context (35–37) and thereby define the specific scope and limitations of the model. Annotation and metadata for this knowledge can comprise additional information, for example a collaboratively curated or consensus strength of evidence and ontologies. These features can provide biological plausibility to those models by design and thus be used to rationalize, explain, and translate representative or individual clinical findings based on the (often large) body of mechanistic knowledge used in the model. Where parameters cannot informed by knowledge and remain unknown, heterogenous (*in vitro*, preclinical, omics, clinical) data can be used for (algorithmic) calibration (38).

The adoption and use of mechanistic models in model informed drug development and especially in regulatory decision making requires to establish their credibility through verification, validation and uncertainty quantification for which existing guidelines need to be adopted by modelers and more specific guidance issued by regulators (34).

In response to the COVID-19 pandemic, mechanistic models have been put forward to guide antiviral drug repurposing (39) and vaccine development (40), showing that such models can synthesize and translate the body of biological knowledge into a clinically relevant setting in a short time frame.

Mechanistic models are associated with a Virtual Population (VPop) to introduce interpatient variability. A VPop is a set of virtual patients, each one being characterized by its own set of descriptors (model parameters values) that follow pre-defined joint distributions (41–43). Simulations can be conducted in varying scenarios (such as different treatment regimens) according to a simulation protocol that defines the entire *in silic*o clinical trial. These *in silico* trials produce digital evidence to explain, complement or partially replace *in vivo* clinical trials for drug development (44, 45). Running mechanistic model based *in silico* trials with a theoretically infinite number of patients can support evidence in rare settings and place population-level results in relation to individual simulated patients. The mechanistic and individual nature of the underlying model further allows one to allocate “clones” of the same patients in different arms and simulation scenarios corresponding to idealized clinical trial settings. In this way, effectiveness can be rationalized through tracing it to impacting and confounding factors.

Mechanistic models thus can bring biological plausibility, equity of clinical and mechanistic evidence as well as individual predictions (similar to idealized RCT settings) to the table of evidence synthesis and generation.

## Modeling in Health Technology Assessment

Modeling in HTA is conducted during (1) the evidence synthesis phase and (2) economic impact assessment, mostly through data-driven modeling approaches. Mechanistic models are still underrepresented in this field but coming of age.

For evidence synthesis, different data-driven modeling approaches are commonly used. Pairwise and network meta-analyses (NMA) (46) using fixed effect and random effects models are tools to synthesize evidence from randomized controlled trials. NMA allows for comparisons that have not been directly obtained in head-to-head trials but comes with methodological challenges. NMA relies on the assumption that the analyzed studies are similar in all factors affecting the relative effects, which can lead to biased results. Moreover, these types of models are often limited in their data source scope. To address this issue, a technique combining NMA with quantitative modeling of effect modifiers (e.g., doses) has become available—utilizing the “totality of evidence” (47). Such “model-based” NMA can mimic randomization and allows estimation and predictions for multiple agents and a range of doses, using plausible physiological dose-response models (48). Additional to data from RCTs, data from observational studies is increasingly used in the evidence synthesis, which, however, lacks an unbiased control arm and techniques for reducing biases need to be applied (49).

For extrapolating a clinical effect into longer-term economic impact there exists quite a variety of methods, which are used for HTA and can be classified as cost-benefit analysis, cost-effectiveness analysis, and cost-utility analysis (50, 51). Simple graph-based decision trees, Markov models [suited for diseases that involve an ongoing risk (52)] or more involved discrete event simulation (DES) (53) and agent-based models (54) are frequently used for data analysis, classification and interpolation and extrapolation in time. The data fed into these models, however, is incomplete due to the limited evidence generated in clinical development (see open questions in Table 1).

Mechanistic models can bridge the gap between development and HTA. Given that validation can establish the credibility of a model for regulatory decision making, exploration of a much larger number of situations than in RCTs (with different patient subgroups, treatment compliance or comparators for instance) might be feasible. Such digital evidence supporting RCT data alleviates several difficulties such as power, representativeness, costs to run the trials, and ethical issues. For the consideration of such evidence in HTA one should consider the following unique benefits of mechanistic model that statistical ones cannot provide. First, mechanistic models possess biological plausibility by design—using biological, chemical, and physical processes as “blueprint”—and are therefore well suited for extrapolations. Second, the VPop can be set up to assess the very same patient under various conditions and scenarios (such as treatment arms) which corresponds to an idealized crossover design and allows to assess clinical benefit for every individual.

A concrete list of examples of how mechanistic models can address unanswered questions left in the MA dossier is given in Table 1. In summary an individual estimate of the (real and long-term) benefit-risk ratio using mechanistic models and adequate metrics (21, 22) feed a precise estimate of the costs of treatment for better health economic projections.

## Modeling for Stakeholder Engagement

HTA is a multi-stakeholder activity that should shed light on more facets of an MP than just a technical analysis. Especially in the appraisal phase, “complex calculations, arbitrary assumptions, debatable choices of whose perspectives to pursue, difficult-to-understand methods, research designs and underlying philosophy/concepts, and time-consuming processes” are at risk of narrowing the HTA findings (55). It has therefore become clear that a diverse set of views need to be captured, consulted, and considered. At the same time, different stakeholders have unique needs that must be addressed before these stakeholders can position themselves. It is to note that recently the importance of engaging patients and patient groups in HTA has been emphasized (56) and there are examples of such engagement in several countries. Nevertheless, systematic involvement from beginning to end of the HTA process [not only during the appraisal stage as currently often the practice (57)] is still an ongoing effort (58). Apart from the need to include different stakeholder groups, there is no consensus what role each stakeholder group should assume in overall decision-making process ranging from information, consultation, participation in the debate, co-decision, as sole decision maker (59). Despite this ongoing debate on the exact role, better mutual understanding, communication, and engagement are sought, all centered around the available evidence. Modeling and simulation and especially mechanistic models may be used as a tool for stakeholder engagement apart from their capability to create (digital) evidence and synthesize data. There is an example from the literature underlining that participation can be achieved by applying an adapted conceptual framework for the modeling and simulation process [see for example (60)]. For this reason, we attempt a mapping of the differences between roles and contributions of stakeholders with specific needs and a suggested use of mechanistic models in Table 2.

**Table 2:** List of different Stakeholder groups increasingly involved in the appraisal stage of HTA with dedicated contribution, special needs (to understand and capture a drug's mechanism, effect, role, or impact) and example of how mechanistic modeling can help to address this need and fill persistent gaps.

**Stakeholder group**	**Contribution to HTA**	**Needs**	**Role of mechanistic models for increasing stakeholder involvement**
**Individual patients or disease-specific citizen and/or patient organizations/associations or caregiver and family member groups**	First-hand experiential knowledge of living with a particular health condition; experience with the health technology under assessment, or currently available technologies, the use of associated health services, and associated benefits, risks, and side effects	Needs to understand the impact of a new MP on personal and individual health status, personal risks, and benefits	Establish plausibility and interactivity of clinical decision-making Highlight potential individual consequences from clinical decision making Highlight individual patient contribution to outcomes (e.g., compliance)
**Citizen and health system user organizations not specific to any condition or disease. Public in general**	May lack knowledge about disease or health technology in question but can assess transparency, legitimacy, and fairness in decision making (61)	Needs to understand reasoning in the decision-making process	Establish plausibility and interactivity of the policy decision-making
**Healthcare professionals Organizations of healthcare professionals**	Gather expertise on clinical aspects regarding: the disease/condition; medical needs; available therapies; the technology under assessment	Needs to be convinced about the new health technology being the best therapeutic approach to be delivered to a patient.	Provide clinically relevant scenarios of HT impact on outcomes, among other comparator approaches
	Identify clinically relevant patient population (and/or subgroups), comparators, thresholds for improvement	Needs to decide, diagnose, or prescribe based on large and complex scientific knowledge	Provide a comprehensive view of all the available scientific knowledge
	Gather information on clinically relevant outcomes including possible neglected outcomes		
	Gaining further information on the importance of outcomes from a healthcare professional point of view (62)		
**Policymakers**	Can judge the expected benefit for healthcare on a national or regional level given the specific political background (63)	Need to estimate a new treatment impact on a national or regional level	Provide trustworthy estimation of a new treatment benefit on a specific population where little data is available
**Payers**	Contribute expertise on reimbursement/coverage decisions Can highlight specific national or regional economic background	Need to estimate a new treatment impact on a national or regional level	Provide trustworthy estimation of a new treatment benefit on a specific population where little data is available
**Companies and associations producing health technologies**	Technology manufacturers can take part (as peers) in all discussions and meetings about contributed data to clarify concerns and provide additional information to support coverage of their products (64).	Needs to understand and rationalize questions and concerns vs. specific available data	Show how technology manufacturer's data fits into the overall evidence Highlight technology and product specific properties with respect to reference
**Academics**	Provide cross-disciplinary scientific feedback from public health, economics, ethics, and social sciences	Needs to understand the bigger picture of HT	Provide information for other models and assessments

There are still barriers dissecting modeling in the HTA space and modeling in the clinical development space. These barriers are conceptually similar to the known barriers to bring HTA to policy making (65). Specific barriers delaying the use of mechanistic models in HTA are (i) the lack of an adapted model validation framework for decision-making process in both contexts (MA and HTA), (ii) inconsistent and unclear support of mechanistic models by the involved stakeholders (competent authorities, and stakeholders involved in HTA likewise), (iii) limited use cases with relevance to clinical development and HTA alike, and (iv) absence of appropriate incentives to use mechanistic modeling throughout the MP development lifecycle. The earlier a dedicated modeling strategy will be put in place the greater will be the demonstrated ability to predict a drug's impact, robustness, and credibility. Bringing mechanistic modeling to HTA, and thus the availability of this tool for the stakeholders requires, however, that drug developers, competent authorities and modelers anticipate the use in HTA.

While **drug developers** could generate more HTA-relevant data during Phase III, the resulting pivotal trials would be more complex and risk missing the statistical target. Drug developers should therefore consider mechanistic models to bridge this gap and report HTA-relevant modeling outcomes, validated with Phase III results.

**Competent authorities** will have a special role in facilitating model-based stakeholder engagement. They should issue more precise and dedicated guidance so that more modeling is included in MA. They should intensify the reporting of mechanistic modeling studies in benefit-risk assessment reports.

**The modeler** needs to embrace the fact that non-experts will also be exposed to the (potentially complex) model and its results. There is a lot of work being done concerning the communication and reporting of clinical trial results to patients and the public which are also applicable for simulated trials. There are EU Commission recommendations on the content of a lay summary (wording and layout) its development and dissemination—Good Lay Summary Practice (66). Communication of complex modeling results could profit from adopting such good practice.

## Conclusion

The immediate and urgent unmet need for interventions and prophylaxis during the COVID-19 pandemic has suggested that drugs backed up by little empirical evidence (compared to the non-pandemic context), but a strong mechanistic background can be approved. The implications of this paradigm shift for HTA still need to be fully understood. In this article, we have advocated that mechanistic models can be used to reproduce, support and extrapolate clinical trials and could constitute a new type of evidence. Mechanistic models can provide causal and quantitative links between patient characteristics, personalized/realistic drug regimen or other extrinsic factors and individual benefit—under consideration of alternative treatment scenarios. They can therefore help to overcome barriers for a more quantitative appraisal of clinical data in HTA and they should also be considered to inform and educate special populations and individuals from a bottom-up perspective. Generation and uptake of *in silico* evidence will need more work of modelers, drug developers, and regulators, who will need to endorse and guide the use of mechanistic models early and consequently in the development process. Likewise, special attention will have to be paid to convey the totality of evidence to different stakeholder groups for empowering them to judge and formulate their specific viewpoint on the MP.

## Data Availability Statement

The original contributions presented in the study are included in the article/supplementary material, further inquiries can be directed to the corresponding author.

## Author Contributions

EC, J-PB, AK, EP, JM, and RK wrote the manuscript. All authors discussed and reviewed the manuscript.

## Conflict of Interest

EC, J-PB, RK, MH, EP, and AK are employed by Novadiscovery. The remaining authors declare that the research was conducted in the absence of any commercial or financial relationships that could be construed as a potential conflict of interest.

## Publisher's Note

All claims expressed in this article are solely those of the authors and do not necessarily represent those of their affiliated organizations, or those of the publisher, the editors and the reviewers. Any product that may be evaluated in this article, or claim that may be made by its manufacturer, is not guaranteed or endorsed by the publisher.

## Footnotes

^1^Please note that, depending on the availability of knowledge, mechanistic modeling approaches may combine a fully mechanistic design for well-known processes with simplifications and assumptions or more phenomenological approaches where knowledge gaps exist. Therefore, the more general term Knowledge-Based Models, (KBM) might be more accurate than “mechanistic models,” but the latter is more widely used.
